# Maternal mRNA input of growth and stress-response-related genes in cichlids in relation to egg size and trophic specialization

**DOI:** 10.1186/s13227-018-0112-3

**Published:** 2018-12-01

**Authors:** Ehsan Pashay Ahi, Pooja Singh, Laurène Alicia Lecaudey, Wolfgang Gessl, Christian Sturmbauer

**Affiliations:** 1 Institute of Biology, University of Graz, Universitätsplatz 2, 8010 Graz, Austria; 2 Evolutionary Biology Centre, Uppsala University, Norbyvägen 18A, 75236 Uppsala, Sweden

**Keywords:** Haplochromine cichlids, Maternal mRNA, Eggs, Trophic specialization, Adaptive radiation, East African lakes

## Abstract

**Background:**

Egg size represents an important form of maternal effect determined by a complex interplay of long-term adaptation and short-term plasticity balancing egg size with brood size. Haplochromine cichlids are maternal mouthbrooders showing differential parental investment in different species, manifested in great variation in egg size, brood size and duration of maternal care. Little is known about maternally determined molecular characters of eggs in fishes and their relation to egg size and trophic specialization. Here we investigate maternal mRNA inputs of selected growth- and stress-related genes in eggs of mouthbrooding cichlid fishes adapted to different trophic niches from Lake Tanganyika, Lake Malawi, Lake Victoria and compare them to their riverine allies.

**Results:**

We first identified two reference genes, *atf7ip* and *mid1ip1*, to be suitable for cross-species quantification of mRNA abundance via qRT-PCR in the cichlid eggs. Using these reference genes, we found substantial variation in maternal mRNA input for a set of candidate genes related to growth and stress response across species and lakes. We observed negative correlation of mRNA abundance between two of growth hormone receptor paralogs (*ghr1* and *ghr2*) across all haplochromine cichlid species which also differentiate the species in the two younger lakes, Malawi and Lake Victoria, from those in Lake Tanganyika and ancestral riverine species. Furthermore, we found correlations between egg size and maternal mRNA abundance of two growth-related genes *igf2* and *ghr2* across the haplochromine cichlids as well as distinct clustering of the species based on their trophic specialization using maternal mRNA abundance of five genes (*ghr1*, *ghr2*, *igf2*, *gr* and *sgk1*).

**Conclusions:**

These findings indicate that variations in egg size in closely related cichlid species can be linked to differences in maternal RNA deposition of key growth-related genes. In addition, the cichlid species with contrasting trophic specialization deposit different levels of maternal mRNAs in their eggs for particular growth-related genes; however, it is unclear whether such differences contribute to differential morphogenesis at later stages of development. Our results provide first insights into this aspect of gene activation, as a basis for future studies targeting their role during ecomorphological specialization and adaptive radiation.

**Electronic supplementary material:**

The online version of this article (10.1186/s13227-018-0112-3) contains supplementary material, which is available to authorized users.

## Background

Parental investment is an important determinant of reproductive success that increases the fitness of the offspring at the cost of parental fitness [1]. The evolutionary trade-off between the two results in parent–offspring evolutionary conflict, which is a cornerstone of kin selection theory [2]. Parental investment is found across the broad taxonomic range of the animal kingdom and can be biparental or exclusively uniparental. It can be divided into two main categories: mating investment and rearing investment. In oviparous organisms, such as fish, maternal mating investment is significantly determined by egg size and clutch size trade-off, optimally modulated by environmental conditions [3]. This investment can affect traits functionally, developmentally, physiologically related to offspring size and survival [4] and also influence later life stages [5]. Thus, the maternal phenotype and the environment she experiences hold adaptive value as they can have important implications for the fitness of offspring.

Egg size is a life-history trait that affects both maternal and offspring fitness, simultaneously [6]. Egg size is determined by a complex interplay of long-term local adaptation and short-term plasticity balancing egg size with brood size [4, 7, 8]. This makes it an evolutionarily important maternal effect [4, 9]. During the female reproductive cycle in oviparous fishes, multiple stages with similar basic patterns occur, such as follicular growth, oocyte maturation and ovulation as well as secondary growth due to incorporation of yolk material into the oocyte (vitellogenesis) [10]. Changes in oocyte growth lead to differences in egg size, which is an important indicator of female energetic investment in reproduction and subsequently affect the development, growth, behaviour and fitness of the offspring [11–19]. A number of interconnected factors are implicated in variation of egg size including female age, size, fecundity, energy acquisition and batch sequence at the time of egg production [20–24] as well as environmental stressors like population density, water temperature, food availability, oxygen level, osmotic changes and predation risk [16, 25–31]. Life-history theories postulate that female investment in offspring quality is balanced with their risk of survival [9]. The molecular mechanisms linking environmental adaptation, e.g. adaptive foraging strategies or stress response, and production of eggs variable in size are poorly understood. Importantly, it is also unclear whether eggs from different species with variable sizes carry different levels of maternally deposited mRNA transcripts triggering particular functions in development, growth and trophic adaptation.

Adaptive radiation is the rapid and simultaneous diversification of a lineage into an array of ecologically disparate species [32]. The haplochromine cichlids from Lake Tanganyika (LT), Lake Malawi (LM) and Lake Victoria (LV) represent the most rapid and species-rich adaptive radiations known [33]. The haplochromine cichlids not only exhibit novel and diverse morphological, ecological and behavioural adaptations, but several of these traits exhibit evolutionary parallelism [34]. Thus, they provide an excellent framework for comparative studies on phenotypic evolution in triplicate radiations of varying age (LT 10–12 million years ago/MYA, LM 2.4–5 MYA, LV 15,000–100,000 years) [35]. In addition, the riverine cichlid species in our study are known to be ancestral to the haplochromine lineages of the three lakes [36–38]. Remarkably, cichlids have highly complex breeding behaviours and all cichlids display a variety of parental investment in the form of egg and larvae care [39]. Maternal mouthbrooding is one such key innovation that characterizes the haplochromines and strongly limits the number and size of eggs that can be incubated [7]. This also means that maternal investment in reproduction is high. Interestingly, life-history traits, e.g. egg size and fecundity, in mouthbrooding cichlids were shown to evolve in parallel to different habitats across the 3 radiations [40]. It was also recently reported that various life-history traits such as clutch size and egg mass in a riverine haplochromine, *Astatotilapia burtoni*, were diverging at the population level, suggestive of ‘ongoing’ adaptive radiation [41]. Furthermore, haplochromines have evolved numerous trophic adaptations to acquire different food sources such as algae, snails, insects and shrimp [42]. Since both the requirements of egg incubation and feeding are fitness-related traits that constrain size/shape of the buccal cavity, it is hypothesized that there is an evolutionary trade-off between the two at the species level [43]. This raises the question of whether the observed traits, i.e. the divergence in trophic specialization and variations in the egg size, can be associated with differences in maternally supplied molecules, such as gene transcripts, with functions in environmental adaptation as well as development, growth and morphogenesis. Also it is interesting to know that maternal provisioning through the deposition of mRNA transcripts of genes with relevant functions can explain potential role for non-Mendelian inheritance in producing an evolutionary trade-off across species. Finally, since trophic specialization, egg size, mouth incubation time and size at independence might be correlated, it is to know if mothers are able to control this process by selective deposition of mRNA transcripts regulating embryonic growth.

The contribution of mRNA transcripts from the mother to the egg is a less studied aspect of maternal investment in offspring fitness. Maternal mRNA transcripts are among the main components deposited in eggs, which are not only critical for early embryonic development, but also affect developmental events after activation of zygotic gene transcription (or maternal to zygotic transition stage; MZT) and influence subsequent developmental life-history trajectories [44]. At early stages of development, maternal RNAs control cellular programmes required for crucial events such as cleavage, blastula formation and gastrulation [45–47]. By the initiation of embryonic transcription during MZT up to half of the maternal RNAs are degraded [48, 49]; however, even at the peak zygotic transcription still majority of mRNA transcripts can have maternal origin in fish [50]. Interestingly, a portion of maternal mRNAs can persist in embryos long after the MZT and function beyond this stage during embryonic morphogenesis [51, 52]. Notably, the products of certain maternal mRNAs might themselves control RNA degradation processes and by postponing the degradation time of specific maternal mRNAs exert their effects on later events of embryonic morphogenesis [53]. It is worth emphasizing that the influence of maternal mRNAs on later developmental events after MZT might not necessarily depend on the presence of the mRNA molecules in later stages since their translated products can persist for much longer period. For instance, persisting activity of an enzyme (involved in retinoic acid production) translated from maternally deposited mRNA was found to be essential for pancreas development in zebrafish [54]. In addition, the tuning of earlier developmental patterning by maternal mRNAs triggers a variety of molecular cascades and cellular processes that organize and guide later developmental events long after clearance of maternal mRNAs, as observed for highly interconnected maternal and zygotic control of dorsoventral patterning in zebrafish [55]. Finally, maternally deposited mRNAs might affect the epigenetic programming during development which can later influence a variety of morphogenetic processes [56]. The molecular mechanisms by which maternal mRNAs influence post-MZT, and particularly, late embryonic development and morphogenesis are poorly explored, although a growing number of studies have begun to focus the spotlight on these processes.

In annual killifish, for instance, different splice variants of maternally supplied gene transcripts have been shown to play a role in directing divergent developmental trajectories during somitogenesis and later affecting plastic responses of embryos to environmental stimuli [57]. The level of maternal mRNA transcript for genes determining ventral embryonic fates was found to be important for the morphogenesis of retina and skeletal structures surrounding the eyes long after initiation of zygotic transcription in cavefish embryos [58]. In round goby, the water temperature experienced by the mother before oviposition leads to selective deposition of maternal mRNAs for temperature-responsive genes which contribute to adaptation to temperature changes during and even beyond embryogenesis [59]. Another recent study in rainbow trout has shown that thermal stress in mother regulates maternal mRNA deposition of genes contributing to neural development and the acquisition of neurocognitive function in embryos which can later shape inter-generational behaviour accordingly [60]. The thermal-induced inter-generational adaptation driven by changes in maternal mRNA and egg size is also reported in stickleback [61]. Maternally deposited transcripts of a nuclear receptor gene appeared to be able to regulate the epigenetic programming of development and indirectly influencing a range of morphogenetic processes in brain, heart, eye and skeletal system [56]. Other examples for association of maternal mRNA deposition with later developmental phenotypes in fish include differences in the length of offspring that contain different levels of maternal mRNAs for specific growth-related genes in brook charr [62] and zebrafish [63].

The deposition of maternal mRNA transcripts occurs at different stages of oocyte formation and is thought to be correlated with certain properties of the egg such as size and its fertilization capacity [44]. Inter-species comparison in amphibians has shown that evolutionary increase in egg size can result in changes in patterns of RNA localization in egg and early embryonic development [64, 65]. Moreover, various studies of teleost fishes have addressed the mechanisms linking egg size variation to changes in fecundity, egg number, follicular development and expression of growth-related genes [20, 66, 67]. Interestingly, several stages of the egg formation process are known to be highly conserved across vertebrates [68–71] and cross-species analysis of mature oocytes has identified the maternal deposition of conserved RNA transcripts across chordates [71, 72]. Growing evidence indicates that a stress-mediated molecular pathway, glucocorticoid (GC) signalling, can be a major determinant of egg size, as well as offspring growth and survival capacity in response to environmental changes [31, 73–76]. However, it is unclear if the GC-mediated changes in egg size are also accompanied with differential deposition of maternal mRNA transcripts for related genes which could then explain how the above-mentioned effects of GC pathway activation in mother during oogenesis are conveyed to the offspring. In addition, interconnected signals mediated by growth hormone and insulin-like growth factors, GH/IGF axis, are well known for their role in egg production under a regulatory mechanism of *ghr* expression (cognate GH receptor) [77]. The axis can be self-limiting during oogenesis since GH-dependent activation of IGF can initiate a feedback mechanism controlling GH production [78, 79]. The level of maternal mRNA components of the GH/IGF axis has also been found to be correlated with egg properties and embryo survival [80, 81]. This indicates that distinct activity of a pathway affecting egg properties during oogenesis might coincide with differential deposition of maternal mRNAs for genes related to the pathway extending the effects to offspring.

In this study, we explored the level of maternal mRNA deposition of selected components of GC and GH/IGF pathways in the eggs of 15 haplochromine cichlid fish species with variable egg sizes and trophic specialization. The species included in this study belong to distinct trophic groups covering three independent radiations, Lake Tanganyika (LT), Lake Malawi (LM), Lake Victoria (LV), and riverine species (RV). The objectives of our study were, first, to investigate whether the eggs from closely related cichlid species contain different levels of maternal mRNAs for components of the two crucial pathways, second, to find out if the potential variations in the maternal mRNAs could be associated with differences in trophic niche specialization or/and egg size, and third, to provide technically accurate quantification method for further investigations in this topic using gene expression analysis. Our results provide first cross-species comparisons of maternal mRNA investment in relation to egg size, habitats and trophic niches in fish as well as a repository of validated reference genes with stable mRNA abundances across the eggs, for accurate normalization of qPCR data, for future studies.

## Methods

### Egg sampling and measurements

**Fig. 1:**
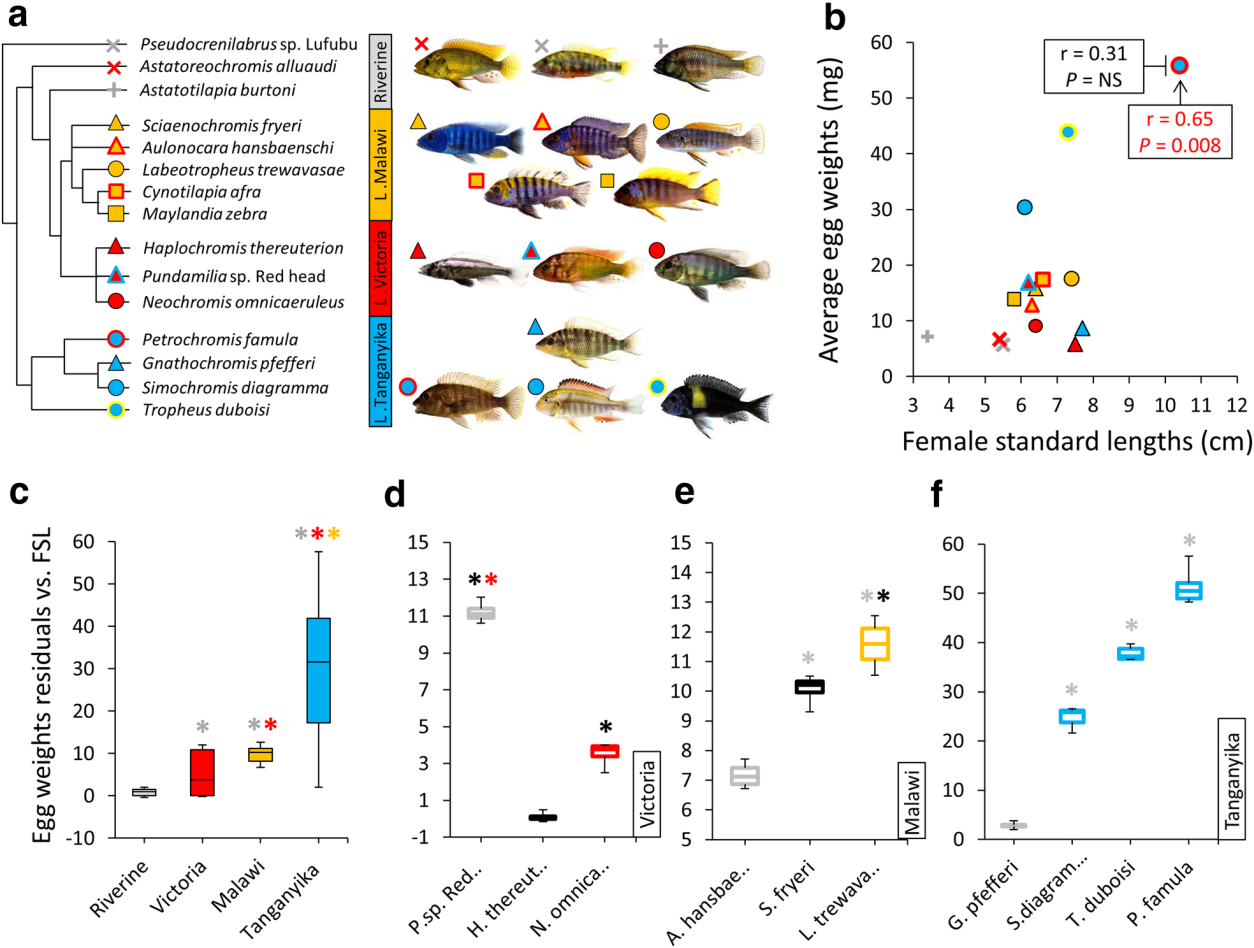
Relatedness, habitats and egg weights of haplochromine cichlid species used in this study. **a** Simplified phylogeny, based on previous genetic studies of East African haplochromine cichlids, representing the relationships between the species included in this study and their habitats. The colours refer to each lake and proximity to one of the lakes for riverine species. **b** Variation in egg weight across the haplochromine cichlid species in respect of the length of female fishes, from which the eggs are produced. **c** Comparison of egg weights, corrected by female standard length, across different habitats. **d**–**f** Comparisons of egg weights, corrected by female standard length, between carnivorous and herbivorous species within each of the three East African lakes. The black and grey enclosed boxes, respectively, indicate piscivorous and invertebrate feeder (carnivorous), whereas other coloured boxes indicate herbivorous species in each lake. Asterisks above box plots indicate significantly elevated expression (*P* < 0.05) compared to the plots matching the colour of the asterisks. In each plot the middle line represents the median and boxes’ lower and upper limits indicate the 25/75 percentiles

In this study we used 15 haplochromine cichlid species, endemic to different habitats in Eastern Africa, including 3 riverine species, 5 species from Lake Malawi, 3 species from Lake Victoria and 4 species from Lake Tanganyika (Fig. 1a). Within each lake, we had at least one herbivorous and one carnivorous species for trophic niche comparison, and a simplified representation of phylogenetic relationships between the species is depicted in Fig. 1a based on previous studies [35, 37]. The fish were raised in separate tanks of mixed-sex species-specific communities (10–14 individuals per tank, with higher ratio of females) with similar light and water conditions and receiving the same diet (Spirulina flakes for cichlids) until young adult stage when mating behaviour was first observed. From this time, we carefully monitored all individuals in each tank on an hourly basis every day, in order to identify any mating pair during the spawning period (up to 3 h). Immediately after the end of spawning, we removed the eggs from the mouth of the females using moderate manual pressure on their cheeks. A single female per species was used for sampling, and from each female up to 8 eggs with no deformities were selected and the females were measured for their standard length (Additional file 2). It should be emphasized that in this study we only look at cross-species comparisons and therefore a single female per species with at least 8 high-quality eggs was used from their first or second batch of laying eggs. Prior to weighing, eggs were checked under microscope for fertilization success and those in stages later than 2-cell cleavage were discarded [82, 83]. One by one, the eggs were quickly dried on a cotton pad in order to remove surface water then weighed to be immediately transferred to RNA*later* solution (Ambion, USA) and kept at − 4 °C overnight. Each egg represented a biological replicate and therefore processed separately, giving a total of 8 replicates per breeding pair and species.

### RNA isolation and cDNA synthesis

Each egg was transferred from RNA*later* solution to a tube with 1.5 mL Qiazol lysis reagent (Qiagen, Hilden, Germany), and a 1.4-mm ceramic sphere was added in the tube to crush the egg. Then, we homogenized the eggs using FastPrep-24 Instrument (MP Biomedicals, Santa Ana, CA, USA) and we extracted RNA based on the protocol developed by the manufacturer suitable for tissues with high fat content. After three washes with 70% ethanol, the RNA was dissolved in 30 µl of nuclease-free and RNase-free water. The genomic DNA contamination was removed using DNase I enzyme (New England Biolabs), and the quantity and quality of each RNA samples were, respectively, checked by Nanophotometer (IMPLEN GmbH, Munich, Germany) and RNA ScreenTapes on an Agilent 2200 TapeStation (Agilent Technologies). First-strand cDNA was synthesized from 500 ng of RNA through High-Capacity cDNA Reverse Transcription kit (Applied Biosystems), following the protocol developed by the manufacturer. The final cDNA product for each sample was diluted 1:4 times in nuclease-free and RNase-free water and was used for qPCR.

### Selection of candidate genes and primer design

We selected 8 candidate reference genes with highest maternal mRNA abundances known in zebrafish eggs, combined with least degradation from the fertilization stage onwards [84]. In addition, we added five more classic reference genes, which are frequently used in studies of different tissues in African cichlids [85–88]. For target genes we selected 6 genes, including two paralogs of growth hormone receptor, *ghr1* and *ghr2*; two insulin growth factors, *igf1* and *igf2*; as well as two components of glucocorticoid pathway, *gr* (also known as *nr3c1*) and *sgk1*.

We designed the qPCR primers matching sections of the sequences that are conserved across East African cichlids, based on transcriptome data from 6 haplochromine species (*Pundamilia nyererei*, *Simochromis diagramma*, *Tropheus duboisi*, *Gnathochromis pfefferi*, *Metriaclima zebra* and *Astatotilapia burtoni*) and two species from more distant tribes (*Oreochromis niloticus* and *Neolamprologus brichardi*) [33, 89]. For this purpose, the coding sequences from all of the above-mentioned species were first aligned in CLC Genomic Workbench, version 7.5 (CLC Bio, Aarhus, Denmark), and then exon/exon junctions were identified based on the annotated genome of *Oreochromis niloticus*, which is available online in the Ensembl database (http://www.ensembl.org) [90]. The primers were designed spanning over these junctions with an amplicon length inferior to 250 bp in order to remove potential effects of possible RNA degradation on qPCR quantification [91]. Primer Express 3.0 (Applied Biosystems, CA, USA) and OligoAnalyzer 3.1 (Integrated DNA Technology) were used to design the primers and for the evaluation of their dimerization and secondary structures.

### qPCR and data analysis

The protocol established by Maxima SYBR Green/ROX qPCR Master Mix (2X) (Thermo Fisher Scientific, Germany) was applied to prepare qPCR reactions, which were conducted in 96-well PCR plates and using ABI 7500 real-time PCR System (Applied Biosystems). Two technical replicates were allocated for each biological replicate following an experimental set-up described as sample maximization method to reach to optimal conditions for a qPCR run [92]. The programme assigned for each qPCR run was initiated with steps of 2 min at 50 °C and a 10 min at 95 °C, continued by 40 cycles of amplification, consisting of 15 s at 95 °C and 1 min at 60 °C. In addition, a dissociation step (60–95 °C) was performed at the end of the amplification step. We also calculated primer efficiency for each primer pair value through LinRegPCR v11.0 program (http://LinRegPCR.nl) [93], and we redesigned primers for pairs showing efficiency values less than 0.9 (Additional file 1).

We used three software packages, with different algorithms, to rank the most stable reference genes: BestKeeper [94], NormFinder [95] and geNorm [96], as well as a ranking based on standard deviation (SD) using raw quantitation cycle (Cq) values. The higher *r* index, in BestKeeper ranking, indicates better stability, whereas in geNorm and NormFinder the lower values, respectively, identified by *M* (average expression stability values) and SV (stability values) suggest more stable reference genes. The average Cq values of the two most stable reference genes were used as normalization factor or Cq_reference_, and for each gene, ΔCq was calculated by subtracting Cq values of the target genes from the corresponding value of the reference genes (ΔCq_target_ = Cq _target _− Cq_reference_). The data from all the egg samples were then normalized to the ΔCq value of a calibrator sample, to obtain a ΔΔCq value (ΔCq_target _− ΔCq_calibrator_). The egg sample with the lowest mRNA abundance (highest ΔCq) was used as a calibrator sample for each gene across all of the species. Relative mRNA quantities (RQ) were calculated based on the expression level of the calibrator sample (E^−ΔΔCq^) [97], and then RQ values were transformed to logarithmic base 2 values (also known as fold differences; FD) [98] for statistical analysis. In order to identify species-specific, lake-specific and trophic niche-specific differences in mRNA abundances, we conducted ANOVA statistical tests, followed by Tukey’s HSD post hoc tests using FD values (Additional file 2). To assess correlation between egg weights and mRNA abundances, Pearson correlation coefficients (*r*) were calculated for each gene in respect of the egg weights across the species. Also, Pearson correlation coefficients (*r*) were used to calculate similarity in patterns of mRNA abundances between all gene pairs. All statistical analyses were implemented in R (http://www.r-project.org).

## Results

### Weight differences of the haplochromine cichlid eggs

The egg measurements across all the species revealed a positive correlation between female length and egg weight; however, this was solely due to a single outlier, the LT species *P. famula*, and the removal of this species from the analysis obliterated the correlation between these variables (Fig. 1b). A comparison of corrected egg weights (using egg weight residuals against female standard lengths) across the lakes showed that LT species had the largest eggs among the selected species followed by LM species with the second largest eggs, while RV species appeared to have smallest eggs (Fig. 1c). In addition, LM and RV species displayed far less variations in egg weight than LT and LV species. Comparisons of egg weight in relation to trophic niche revealed larger eggs for herbivorous than carnivorous species across the lakes except for one of the carnivorous species in LV (Fig. 1d–f); notwithstanding that, more species per trophic niche from each lake are required to confirm this as a general trend in the eggs of haplochromine cichlids.

### Suitable reference genes for quantification of maternal mRNA

**Fig. 2:**
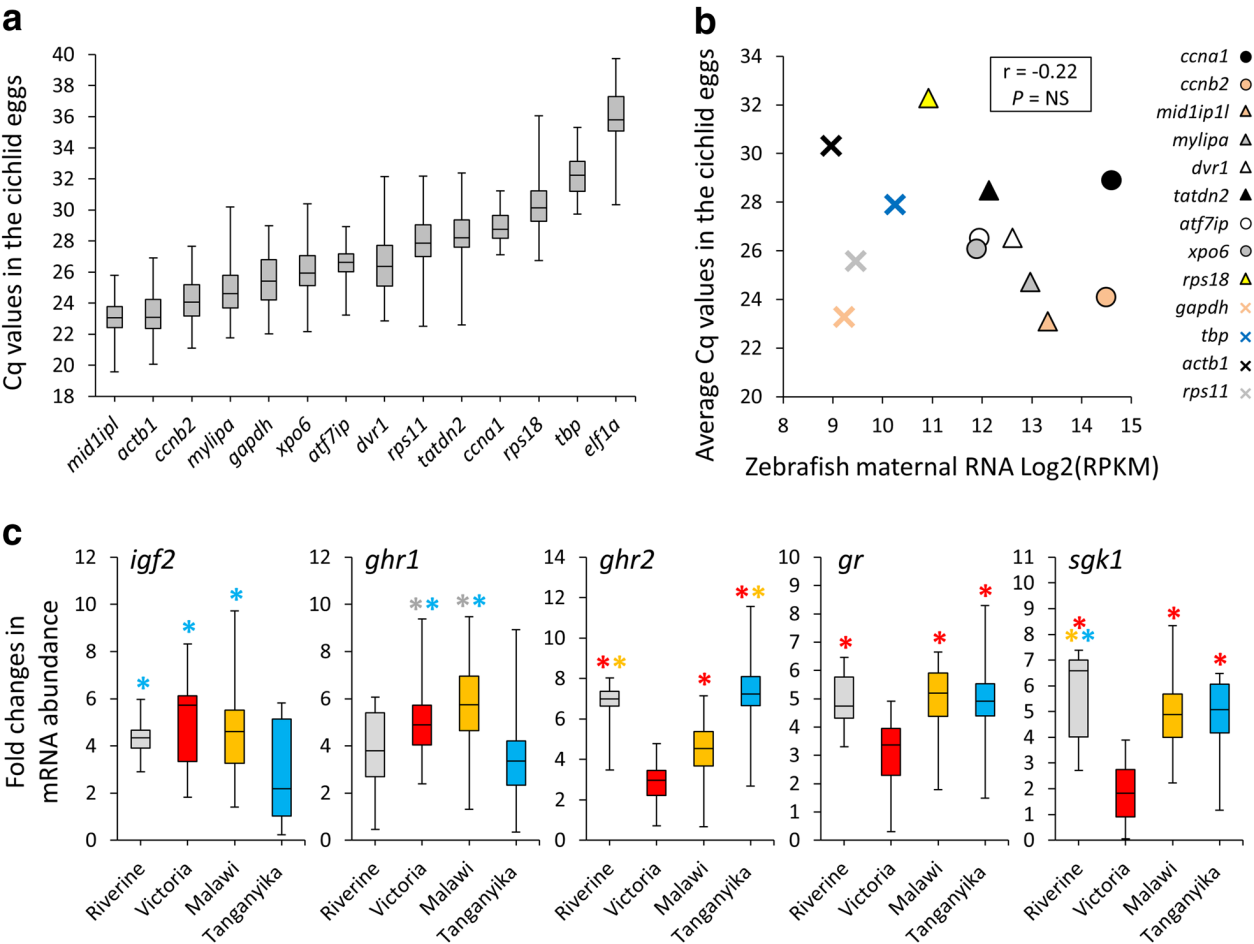
Maternal mRNA abundance of candidate reference and target genes. **a** mRNA abundance of candidate reference genes based on raw Cq values across all of the species. In each box plot, the middle line represents the median and boxes’ lower and upper limits indicate the 25/75 percentiles. **b** Correlation between average mRNA abundance of the reference genes, in eggs of haplochromine cichlids and zebrafish. **c** Comparisons of maternal mRNA abundance for candidate target genes across different habitats in East Africa. Asterisks above box plots indicate significantly elevated expression (*P* < 0.05) compared to the plots matching the colour of the asterisks. The middle line represents the median and boxes’ lower and upper limits indicate the 25/75 percentiles for each plot

In order to accurately measure mRNA abundance in eggs and be able to compare the quantities across the cichlid species, we needed to first identify reference genes with least variations in mRNA abundance among the eggs of different species [99]. To select reference gene candidates, we retrieved transcriptome data available for zebrafish maternal mRNA and identified 8 genes displaying highest mRNA abundances with no significant variations from post-fertilization stage to the initiation of zygotic transcription (mid-blastula stage or MBT which happens at the beginning of MZT stage described above) [84]. The study was conducted on a large number of zebrafish embryos harvested at five different developmental stages: 1-cell, 16-cell stage, 128-cell stage, MBT and post-MBT [84]. In addition, we included 6 more candidates, *actb1*, *ef1a*, *gapdh*, *rps11*, *rps18* and *tbp,* which are shown to have high expression in different tissues and were used as stable reference genes in a variety of studies on African cichlids [85–88]. Expectedly, the genes had a range of mRNA abundances, from highest levels of transcripts for *mid1ip1*, *actb1* and *ccnb2* to lowest levels for *rps18*, *tbp* and *ef1a* (Fig. 2a). When compared to zebrafish transcriptome data [84], we did not find significant correlation between the mRNA abundances of the genes in cichlid and zebrafish eggs (Fig. 2a). However, five genes, *mid1ip1*, *mylipa*, *dvr1*, *tatdn2* and *rps18* showed high to low mRNA abundances, respectively, in the cichlids and zebrafish, which are specified with triangles in Fig. 2a. It should be noted that the higher Cq values indicate lower mRNA abundances in the cichlid eggs, whereas higher RPKMs mean higher mRNA abundances in zebrafish; therefore, the opposite pattern between the two value types indicates similarity in mRNA abundance between the cichlids and zebrafish (Fig. 2a). In addition, *ef1a* with lowest mRNA abundance in the cichlid eggs had also below cut-off level of sequencing reads in zebrafish egg transcriptome [84], which might indicate partial conservation in the levels of maternal mRNA investment for certain genes across distant teleost taxa.

Among the candidate reference genes *mid1ip1* had the lowest standard deviation (SD) in the eggs from different cichlid species followed by *ccna1* and *atf7ip* as second and third most stable genes in this ranking (Table 1). After selecting only the top 8 genes with lowest SD to run on BestKeeper, *atf7ip*, *ccnb2* and *mid1ip1* were ranked as top three genes by this software. NormFinder ranked *atf7ip*, *ccnb2* and *actb1* as the top three most stable genes. On the other hand, geNorm suggested only *atf7ip* and *mid1ip1* as suitable reference genes since their M value was below the recommended threshold (*M* < 1.5). Importantly, each of these two genes alone showed significant differences in at least one of the comparisons of the trophic niches and/or the lakes, but their average Cq values did not show differences in these comparisons (Additional file 3: Figure 1). Hence, we used average Cq value of *atf7ip* and *mid1ip1* in each egg as normalization factor (NF) for further quantifications of mRNA abundance. It should be noted that NF was also ranked as most stable normalizing value when applied in all three ranking algorithms (Table 1).

**Table 1:** Ranking of candidate reference genes across eggs of haplochromine cichlid species using three different softwares

BestKeeper				geNorm		NormFinder	
Ranking	SD	Ranking	*r*	Ranking	*M*	Ranking	SV
NF	0.930	NF	0.805	NF	1.287	NF	0.602
*mid1ip1*	0.954	*atf7ip*	0.781	*atf7ip*	1.344	*atf7ip*	0.615
*ccna1*	0.969	*ccnb2*	0.761	*mid1ip1*	1.405	*ccnb2*	0.688
*atf7ip*	1.112	*mid1ip1*	0.679	*actb1*	1.511	*actb1*	0.688
*actb1*	1.286	*actb1*	0.678	*tatdn2*	1.519	*xpo6*	0.766
*tbp*	1.331	*mylipa*	0.635	*xpo6*	1.522	*mid1ip1*	0.785
*ccnb2*	1.500	*rps18*	0.617	*ccnb2*	1.522	*tatdn2*	0.814
*rps18*	1.566	*tbp*	0.592	*gapdh*	1.528	*mylipa*	0.815
*mylipa*	1.575	*ccna1*	0.485	*ccna1*	1.592	*ccna1*	0.900
*xpo6*	1.587	*xpo6*	–	*tbp*	1.634	*gapdh*	0.903
*tatdn2*	1.630	*tatdn2*	–	*mylipa*	1.653	*tbp*	0.911
*gapdh*	1.655	*gapdh*	–	*rps18*	1.752	*rps18*	1.028
*elf1a*	1.692	*elf1a*	–	*dvr1*	1.771	*dvr1*	1.042
*rps11*	1.991	*rps11*	–	*rps11*	1.943	*elf1a*	1.050
*dvr1*	2.045	*dvr1*	–	*elf1a*	2.044	*rps11*	1.134

*SD* standard deviation, *r* Pearson product-moment correlation coefficient, *SV* stability value, *M* M value of stability, *NF* normalization factor, indicating geometric mean for Cq values of *atf7ip* and *mid1ip1* genes

### Maternal mRNA abundance of candidate target genes

The mRNA abundance of 6 target genes, *igf1*, *igf2*, *ghr1*, *ghr2*, *gr* and *sgk1*, was examined across the eggs, and we found *igf1* transcripts to be below the minimum detection level through qPCR in all of the species in this study. The lake-specific comparisons of mRNA abundances revealed lower mRNA investment of *igf2* in LT with largest eggs (Fig. 2c). We also found lower mRNA abundance of *ghr2*, *gr* and *sgk1* in LV compared to the other habitats. In contrast, *ghr1* showed higher mRNA abundance in LV and LM compared to LT and RV. These results reveal clear variations in maternal mRNA investment for all of the candidate target genes, depending on the habitats of closely related haplochromine cichlid fishes. Moreover, the two paralogs of a growth hormone receptor, *ghr1* and *ghr2*, displayed opposite expression patterns, reflecting contrasting maternal mRNA investments, which suggests lake-specific divergence of their role during early development. The lower transcripts of *gr* and *sgk1* might imply distinct activation of GC pathway in LV species during deposition of maternal mRNA in the eggs.

**Fig. 3:**
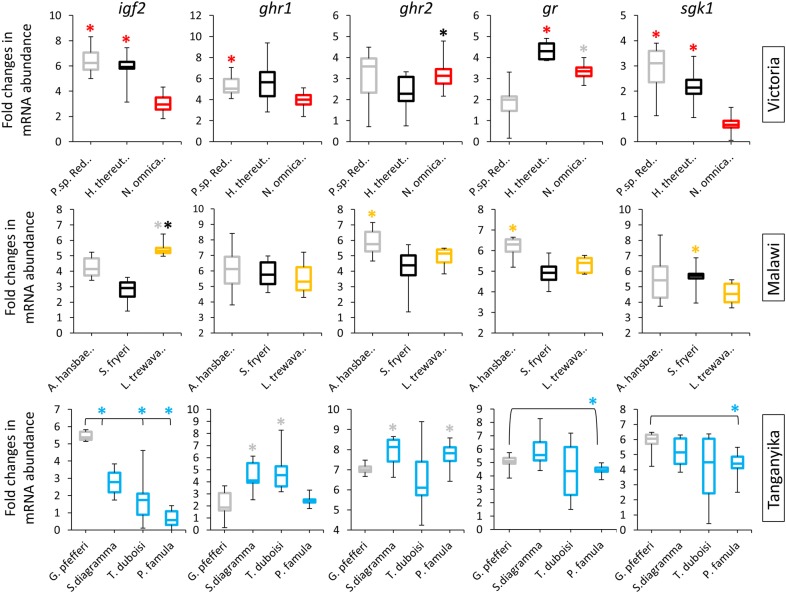
Maternal mRNA abundance of candidate target genes compared between carnivorous and herbivorous species. Comparisons of maternal mRNA abundance for candidate target genes between carnivorous and herbivorous species within each lake. The black and grey enclosed boxes, respectively, indicate piscivorous and invertebrate feeder, whereas other coloured boxes indicate herbivorous species in each lake. Asterisks above box plots indicate significantly elevated expression (*P* < 0.05) compared to the plots matching the colour of the asterisks. The middle line represents the median and boxes’ lower and upper limits indicate the 25/75 percentiles for each plot

The comparisons between herbivorous and carnivorous species revealed differences in mRNA abundance of the target genes in each lake (except for *ghr1* in LV) (Fig. 3). A similar pattern of higher mRNA abundance was observed between a carnivorous and herbivorous species for *ghr2* and *gr* in LM, and for *gr* and *sgk1* in LT. Furthermore, a consistent pattern of higher mRNA abundance in carnivorous species was observed for *igf2* in LV and LT, but this pattern was reversed in LM (with higher level of transcripts in herbivorous species). Among the target genes, only *sgk1* showed a tendency towards higher mRNA abundance in carnivorous species across all lakes; however, the difference was not statistically significant in all of the species with contrasting trophic niches.

### Correlation patterns of target gene maternal transcripts

**Fig. 4:**
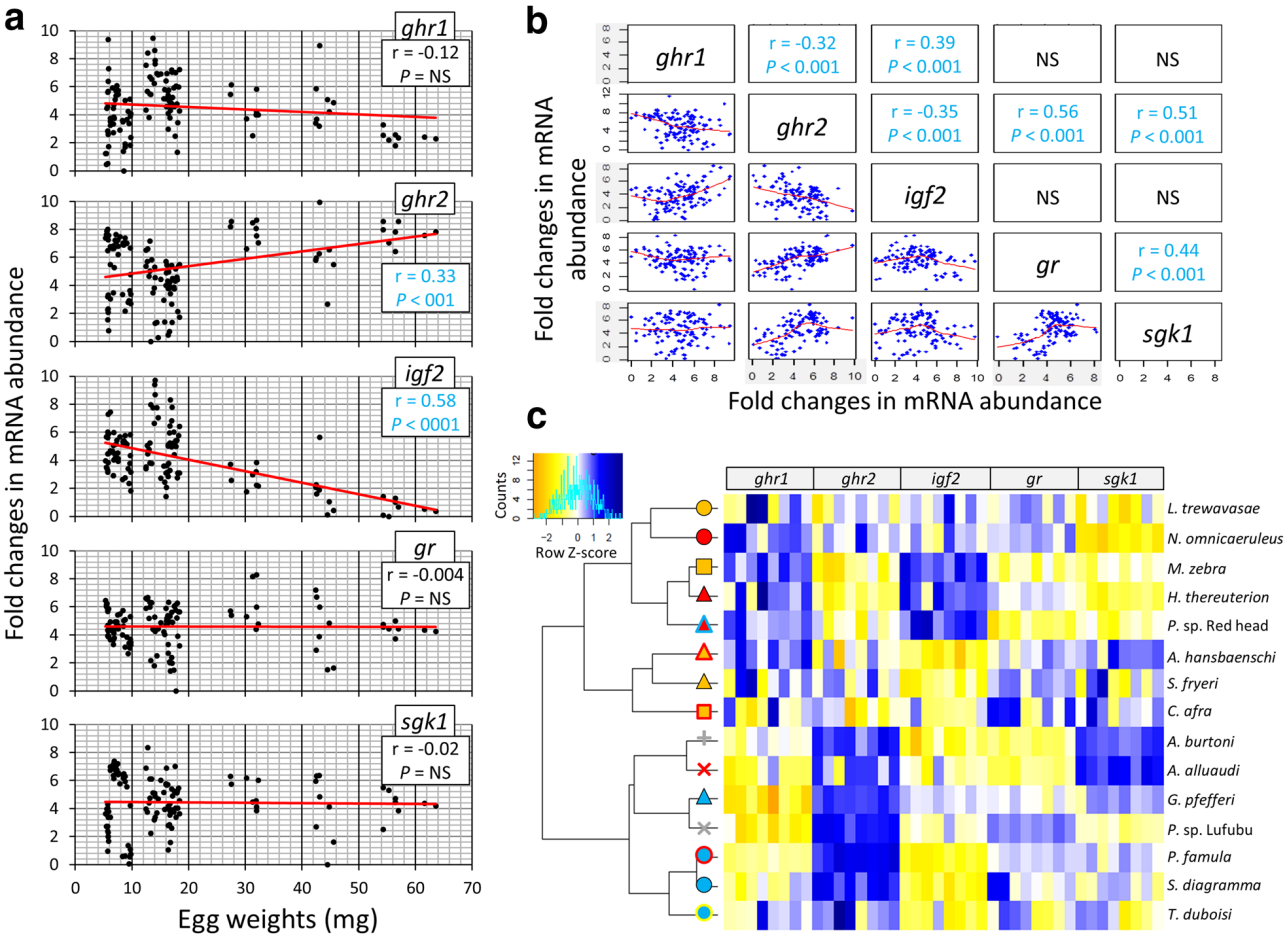
Correlation analyses of maternal mRNA abundance of candidate target genes across the haplochromine species used in this study. **a** Pearson correlation coefficient (*r*) was used to assess the similarity between differences in mRNA abundance of the target genes and egg weights across all species. **b** Pearson correlation coefficient (*r*) analyses, assessing the pairwise expression similarity between the candidate target genes. **c** A dendrogram clustering species based on expression pattern similarities of the candidate target genes, when combined together. The species are represented by the same symbols used in Fig. 1a

We assessed the correlations between maternal mRNA abundance of the target genes and egg weights across all the haplochromine species in this study. Interestingly, the mRNA abundance of two genes, *ghr2* and *igf2*, displayed significant positive and negative correlations with egg weights, respectively (Fig. 4a). This indicates inter-species differences in maternal mRNA deposition of the two growth-related genes, with respect to the egg mass in cichlids. Next, we conducted pairwise comparisons of mRNA abundance between the target genes in order to identify potential transcriptional regulatory connections linking the genes (Fig. 4b). Expectedly, we found a positive expression correlation between the mRNA abundances of *gr* and *sgk1,* which both belong to GC signalling pathway, confirming the accuracy of our qPCR analyses. Furthermore, both *gr* and *sgk1* mRNA abundances appeared to have positive correlations with *ghr2* mRNA abundance (but not with *ghr1*), suggesting a potential regulatory connection between *ghr2* and activated GC pathway in the cichlid eggs. In contrast, *ghr2* displayed negative correlation with *igf2* and *ghr1* mRNA abundances, while *igf2* and *ghr1* had a positive correlation across the eggs. These might be the result of transcriptional decoupling between *ghr2* and GH-IGF regulatory axis due to a mechanism affecting only *ghr2* transcription and maternal deposition.

Finally, we performed a hierarchical clustering of the target genes based on their mRNA abundances comparing the relationships between the species (Fig. 4c). Strikingly, the dendrogram showed the presence of two main clusters dividing LV and LM species from RV and LT species, implying similar patterns of maternal mRNA abundance in LT and RV species, while they are both distant from the other two lakes. In RV–LT cluster, the three LT herbivorous species (*T. duboisi*, *P. famula* and *S. diagramma*), with larger eggs, were sub-clustered together, whereas the LT carnivorous species (*G. pfefferi*), with smaller eggs, was sub-clustered with RV species. Two sub-clusters were also observed in LV–LM cluster, and in one sub-cluster, three of the LM species were grouped together, whereas in the other sub-cluster, the three LV species grouped with two other LM species. A closer look into the branching pattern of the dendrogram revealed that the two LM carnivorous species (*A. hansbaenschi* and *S. fryeri*) with an LM omnivorous species (*C. afra*) constitute one of the sub-clusters. Interestingly, in the other sub-cluster, the herbivorous species of both LM and LV (*L. trewavasae* and *N. omnicaeruleus*) are grouped together, whereas the two LV carnivorous species (*P.* sp. *Red head* and *H. thereuterion*) and a LM omnivorous species (*M. zebra*) constitute another group. These observations demonstrate that, based on maternal mRNA abundances of only the five target genes, a clear distinction is observed between LT and RV versus LM and LV. In addition, within each cluster a distinct grouping between herbivorous and carnivorous species is observed for each lake.

## Discussion

Differences in the level of maternal mRNA transcripts between eggs can be a result of distinct maternal provisioning for mRNA deposition during oogenesis. On the other hand, the amount of mRNA transcripts in eggs starts to change soon after fertilization and substantially declines from the initiation of zygotic transcription (mid-blastula transition) due to degradation process affecting most maternal gene transcripts [84]. In zebrafish, a proportion of the maternal mRNA appeared to be degraded prior to mid-blastula transition (~ 3.5 h post-fertilization; hpf) [84]. It should be noted, however, that the maternal mRNAs still account for a significant part of functional mRNA transcripts during zygotic transcription and some could persist in later stages of development as well (see introduction). The great diversity of cichlid fishes in trophic specialization, manifested in distinct morphology, growth, developmental patterning, egg size, provides an excellent model system to investigate potential links between maternal mRNA provisioning, life history and ecomorphology. Such studies can provide insight into the role of life-history traits and maternal effects in adaptive radiations. In cichlid fishes studied so far, the developmental stage comparable to mid-blastula transition occurs later in time than zebrafish, e.g. around 12 hpf in two African cichlids, *Oreochromis niloticus* and *Astatotilapia burtoni* [82, 100], and 4 hpf in two American cichlids, *Symphysodon aequifasciatus* and *Amphilophus spp.* [83, 101]. Therefore, we set out to investigate the maternal mRNA investment in cichlid eggs almost immediately after fertilization (up to 3 hpf) to minimize the effects of RNA degradation during early development. This early stage of cichlid embryogenesis also has implications for the developmental hourglass hypothesis where species are evolutionarily most divergent at the early and late stages of embryonic development, with a conserved stage in between [102].

### Importance of reference genes for accurate quantification of maternal mRNA abundances in eggs

In order to accurately quantify maternal mRNA abundance of our target genes of interests we had to identify reference gene transcripts showing no signs of degradation in the early developmental stages. This is of paramount importance when studying relative gene expression in different tissues. Among the candidate reference genes, which were selected based on their resistance to RNA degradation and high level of maternal transcripts in zebrafish eggs [84], we found the cichlid homologous of two genes, *atf7ip* and *mid1ip1*, to have the most stable level of mRNA abundance across the eggs of haplochromine cichlids. The first gene, *atf7ip* (also known as *mcaf1*), encodes a transcriptional modulator participating in histone methylation process and chromatin organization [103] and contributes to the maintenance of X chromosomes inactivation during female cell development and division in mammals [104]. In bovine oocyte, *atf7ip* has been implicated in the repression of oocyte-specific genes when the embryonic gene expression is beginning to take place [105]. It is worth emphasizing that the maternal methylation imprinting can play an important role both during oogenesis and in later stages of embryonic development [106, 107]. In our study, the consistent mRNA abundance of *atf7ip* across all of the haplochromine species suggests its tight regulation during final stages of oogenesis when the maternal mRNA deposition takes place and its transcript stability in early development.

The second validated reference gene, *mid1ip1* (*mig12*), encodes a protein required for efficient lipid biosynthesis, stabilization of microtubules in cytoskeleton and embryonic midline development [108, 109]. In zebrafish a mutation in this gene has demonstrated that the maternally provided *mid1ip1* transcripts are crucial for cytoskeletal dynamics and membrane recycling in early cell divisions, and during the reorganization of the cytoskeleton at the egg-to-embryo transition [110]. Therefore, the observed low variation in abundances of *mid1ip1* transcripts across the eggs implies its tight regulation during late oogenesis, uniform maternal deposition across species and potential requirement in early development of the cichlid species. In addition to these candidate genes, we also tested the maternal abundance of 5 classic reference genes, frequently used in different studies of cichlid tissues, and we found that none of them appeared among the top validated reference genes with different ranking methods and therefore were not suitable for analysis of qPCR data obtained in this study. This once again underscores the necessity of careful validation of reference genes in each qPCR study, according to different experimental conditions, tissues and species [111]. The reference genes validated here will be a useful resource for future studies on this topic.

### Egg weight may be an indicator of adaptive divergence in cichlids

Given the importance of egg size as an indicator of maternal investment and life-history strategies, we were interested in investigating the relationship between mRNA abundance of a selected set of target genes, involved in early developmental growth and mediating the effects of environmental signals, and the egg size in haplochromine cichlids. Therefore, we first had to characterize the eggs based on their variations in weights across habitats and trophic niches. Indeed, the smallest eggs were found in the riverine haplochromines, while the largest eggs were observed in species of the oldest species flock in Lake Tanganyika. Also herbivorous species tended to have larger eggs than carnivorous species. However, the number of species with contrasting trophic niches per lake was not enough in our study to conclude with confidence that the observed differences are consistent throughout all haplochromine cichlids. A plausible explanation for the increase in egg size in all lake habitats, as well as for the increase with evolutionary age, might be adaptation to more predictable niches in lake habitats, as opposed to much more fluctuating environmental parameters in riverine environments.

### Species of the youngest lake deposit the lowest amount of maternal mRNAs for stress-mediating genes

The glucocorticoid (GC) pathway can be considered as a prime candidate molecular signal affecting processes involved in ovulation, egg size determination, offspring growth and survival in response to environmental changes [31, 73–76, 112–117]. Concerning the target genes in our study, we examined the maternal mRNA abundance of two components of GC signalling pathway: a nuclear receptor, *gr* (or *nr3c1*), which also acts as an upstream transcriptional regulator of GC pathway, and a downstream effector of the pathway, *sgk1*, which is a kinase activating certain ion channels and mediating cellular stress response [118–120]. The activity of enzyme encoded by *sgk1* is regulated by mTOR signalling pathway [121, 122], which is a modulator of oogenesis (primordial follicle activation and arrest) [123]. In this study, we found a positive correlation between mRNA transcript abundances of *gr* and *sgk1* genes across all the eggs confirming their regulatory connection during early development, prior to mid-blastula transition. Moreover, both genes had very similar patterns of transcript abundance across the lakes with lowest level of abundance in LV. This proposes a potential difference in response to environmental stressors, which can be reflected in maternal deposition of GC pathway gene transcripts, and possibly, their distinct effects on early development between the cichlid species in LV and those in the other habitats. Differences in *sgk1* expression have been already suggested as an adaptive response to environmental stressors in vertebrates [124, 125], and for instance, it can be directly regulated by osmotic changes [126]. This might be a highly conserved mechanism linking environmental cues to maternal provisioning since *sgk1* has been found to be a regulator of fat storage (in eggs), embryonic growth and stress response in *Caenorhabditis elegans* as well [127, 128]. The maternal *gr* transcripts have been shown to be essential for developmental programming of different tissues, in particular for skeletal system development in zebrafish embryos [53]. Thus, it would be interesting to know whether LV cichlid species display distinct skeletogenesis during embryonic development than other haplochromine species in the other lakes. Although the activation of GC pathway can affect egg size, we did not find any correlation between maternal abundance of *gr* and *sgk1* with egg weight across the species, suggesting that such effect does not necessarily lead to changes in maternal deposition of the GC-related mRNAs, and thus studies of pre-ovulatory stages in the ovary are required to address this role in cichlids.

### Larger eggs contain higher amount of maternal *ghr2*, but not *ghr1* across haplochromine cichlids

There are two growth hormone (GH) receptors in teleost fish, *ghr1* and *ghr2*, and even though both receptors show similarities in signal transduction and transcriptional regulation of certain downstream genes, there is evidence for their tissue-specific function and expression as well [129–134]. In particular, the overall expression level of *ghr2* has been found to be higher than *ghr1* in fish gonadal tissues [77, 131, 135], and during ovary reproduction cycle in cichlid, *ghr2* shows higher expression at sexual recrudescent and regressed stages, whereas at sexual matured stage there is an increase in expression of both receptors without significant difference between them [77]. This could indicate a stage-specific regulatory role of *ghr2* during ovary reproduction cycle, as well as a regulatory role of both receptors in oocyte maturation. In our study, we found higher abundance of *ghr1* maternal transcripts in LV and LM, whereas this pattern was reversed for *ghr2* maternal transcripts (i.e. higher *ghr2* in RV and LT eggs). This suggests a similarity between LT and RV haplochromine species in maternal deposition of *ghr* genes, and a possible evolutionary divergence of LV and LM from this pattern. These results support lineage-specific specialization of *ghr* genes in younger (LM and LV) versus older (LT) and seeding lineages (RV). Interestingly, *ghr1* and *ghr2* displayed negative correlation in maternal mRNA abundance across all the eggs, which can be a result of distinct regulatory effects of upstream factor(s) on their transcription during oocyte maturation and subsequent maternal mRNA deposition. It is already known that several factors with a role in oogenesis, such as cortisol, insulin and GH, also have distinct effects on *ghr1* and *ghr2* transcription in fish [131, 136]. For instance, insulin can repress *ghr2* transcription, while inducing *ghr1* transcription in cichlid liver cells [136]. Furthermore, we found that larger eggs had higher abundance *ghr2*, but not *ghr1*, across the haplochromine cichlids. This is in agreement with the results of an intra-specific study of maternal mRNA investment in a haplochromine species with variable egg sizes in which *ghr* (not specified at the paralog level) had shown higher mRNA abundance in larger eggs [67]. Interestingly, in the same study, individuals from smaller eggs with lower maternal *ghr* abundance later expressed higher *ghr* expression during larval development, a compensatory mechanism through which they could catch up in size with those individuals originating from larger eggs [67]. Future inter-specific studies are required to know whether such a mechanism is ubiquitous in haplochromine cichlids. Our results, together with observations in other studies of GH receptors, suggest inter-specific and egg-size-dependent mechanism underlying maternal *ghr2* transcript deposition which might contribute to adaptive developmental events in cichlids.

### Larger eggs contain lower amount of maternal *igf2* across haplochromine cichlids

Finally, we investigated the maternal investment for two insulin growth factors, *igf1* and *igf2*, across the species. The proteins encoded by these paralog genes are functionally and structurally related to insulin hormone and play diverse roles in mediating growth and development in vertebrate including physiological processes controlling fish reproduction [137]. Both factors, for instance, play a role in the formation of vitellogenic follicles and the promotion of oocyte maturation in fish ovaries [138–141]. The maternal mRNA abundances of *igf1* and *igf2* can differ in fish as greater abundance of *igf2* mRNAs compared to *igf1* was observed in follicles [44, 142] and post-fertilized eggs [143]. Moreover, the oocyte investment of *igf1* mRNA had been shown to be very little across distant teleost fish taxa [137, 142]. Similarly, we found maternal *igf1* abundance to be below the minimum detection level by qPCR across the cichlid eggs, whereas maternal *igf2* mRNA was detected in all of the eggs. We also found lower amounts of maternal *igf2* transcripts in larger eggs which have never been reported in fish, proposing an unknown mechanism during maternal provisioning. To our knowledge, it has only been shown in porcine ovaries that the largest follicles contain lower *igf2* expression, when compared to average or small size follicles [144]; however, the mechanism underlying this expression pattern and its potential impacts on embryonic development remains unknown. We observed negative correlation in mRNA abundance between *igf2* and *ghr2,* which could implicate shared upstream regulator(s) influencing maternal mRNA deposition of both genes in an opposite manner. A candidate for such an upstream regulator could be the insulin signalling pathway, which is among the few signals that oocytes, at early stages of development, are responsive to, and while high levels of insulin can impair oocyte growth, low amounts can, on the contrary, promote oocyte growth [145]. Interestingly, it is already shown that insulin can induce *igf2* expression and repress *ghr2* and *igf1* expression in cichlids [136, 146]. It should be noted that insulin signalling is also involved in yolk formation of fish oocytes, through the regulation of vitellogenesis [147].

### Adaptive trophic divergence may influence maternal mRNA deposition of growth- and stress-related genes in cichlids

Perhaps the most interesting finding of our study might be the clear clustering of species according to trophic niche, based on their maternal mRNA abundances of the target genes, when we combined the data together. For instance, the two carnivore species in each of LM and LV were clustered together, whereas the herbivore species of all 3 lakes were placed as separate clusters. Also, the three herbivore species in LT were clustered in one sub-branch, but the LT carnivore species was placed distantly, closer to the RV branch. Considering that distinct trophic niche clustering was observed using the maternal mRNA abundances of only 5 candidate genes, this could suggest potential predictability in differentiating herbivore and carnivore cichlid species based on their maternal mRNA investment. This points to disparate adaptive trajectories, which might influence subsequent developmental pathways in species specialized to a particular trophic niche. It should be noted that the haplochromine cichlids, with different trophic specializations, possess morphologically distinct skeletal structures features in dentition and jaw bones, which play an important role in their adaptive divergence [33, 148–152]. Our findings support the idea of the parallel evolution at the molecular level of trophic similar trophic niches and maternal mRNA investment across the parallel cichlid adaptive radiations [40]. However, a more in-depth analysis of more genes and species is necessary to substantiate this claim. Knowing that the two signalling pathways, GC and IGF, are both involved in trophic skeletal formation during early development in fish [152], and particularly since recent studies in haplochromine cichlids and pupfish raise the possibility of IGF involvement in adaptive craniofacial divergence [153, 154], our study might implicate potential associations between maternal RNA deposition and differential early developmental patterning of diverse feeding apparatus in fishes. This is interesting because the pleiotropic involvement of both pathways in feeding and incubation of eggs could be key to controlling the trade-off between a morphological (jaws and feeding) and behavioural phenotype (mouthbrooding) in haplochromine cichlids [43].

## Conclusions

In this study, we provide first insight into the molecular basis of an important life-history trait, namely by the identification of links between trophic specialization, habitats/lakes and maternal mRNA deposition of specific genes in East African cichlids. Our results show that maternal mRNA inputs vary substantially across cichlid adaptive radiations and with respect to trophic specializations, based on selected key genes involved in growth-related and environmental stress-mediating molecular pathways in association with egg size. We speculate that the evolutionary trade-off between mouthbrooding and feeding may be influenced by the same pleiotropic stress- and growth-related molecular pathways. However, further studies using large-scale transcriptional profiling with higher number of cichlid species with distinct feeding and breeding strategies are required to support this notion. Moreover, our findings shed light on the possible molecular interactions involved during this early and divergent stage of the hourglass hypothesis in cichlids and beyond, as the regulatory mechanisms causing the hourglass pattern still remain open to exploration [155, 156].

## Additional files

**Additional file 1.** Information about qPCR primers used in this study.

**Additional file 2.** Statistical results, raw gene expression data, egg weights and female lengths.

**Additional file 3: Figure** **1.** Differences of maternal mRNA abundance for *mid1ip1* and *atf7i,* as well as geometric means their Cq Values (NF) in comparisons of the lakes (A) and the trophic niches (B). Asterisks above box plots indicate significantly elevated expression (*P* < 0.05) compared to the plots matching the colour of the asterisks. The middle line represents the median and boxes lower and upper limits indicate the 25/75 percentiles for each plot.

## Abbreviations

LT: Lake Tanganyika
LM: Lake Malawi
LV: Lake Victoria
*ghr*: growth hormone receptor gene
*igf*: insulin-like growth factor gene
*gr*: glucocorticoid receptor gene
*sgk1*: serum/glucocorticoid-regulated kinase 1 gene
GC: glucocorticoid pathway
IGF: insulin-like growth factor pathway
