# Supplementary figures and images for: Maternal mRNA input of growth and stress-response-related genes in cichlids in relation to egg size and trophic specialization
Source: EvoDevo. 2018 Dec 1;9:23. doi: 10.1186/s13227-018-0112-3 (PMC6271631; doi:10.1186/s13227-018-0112-3)

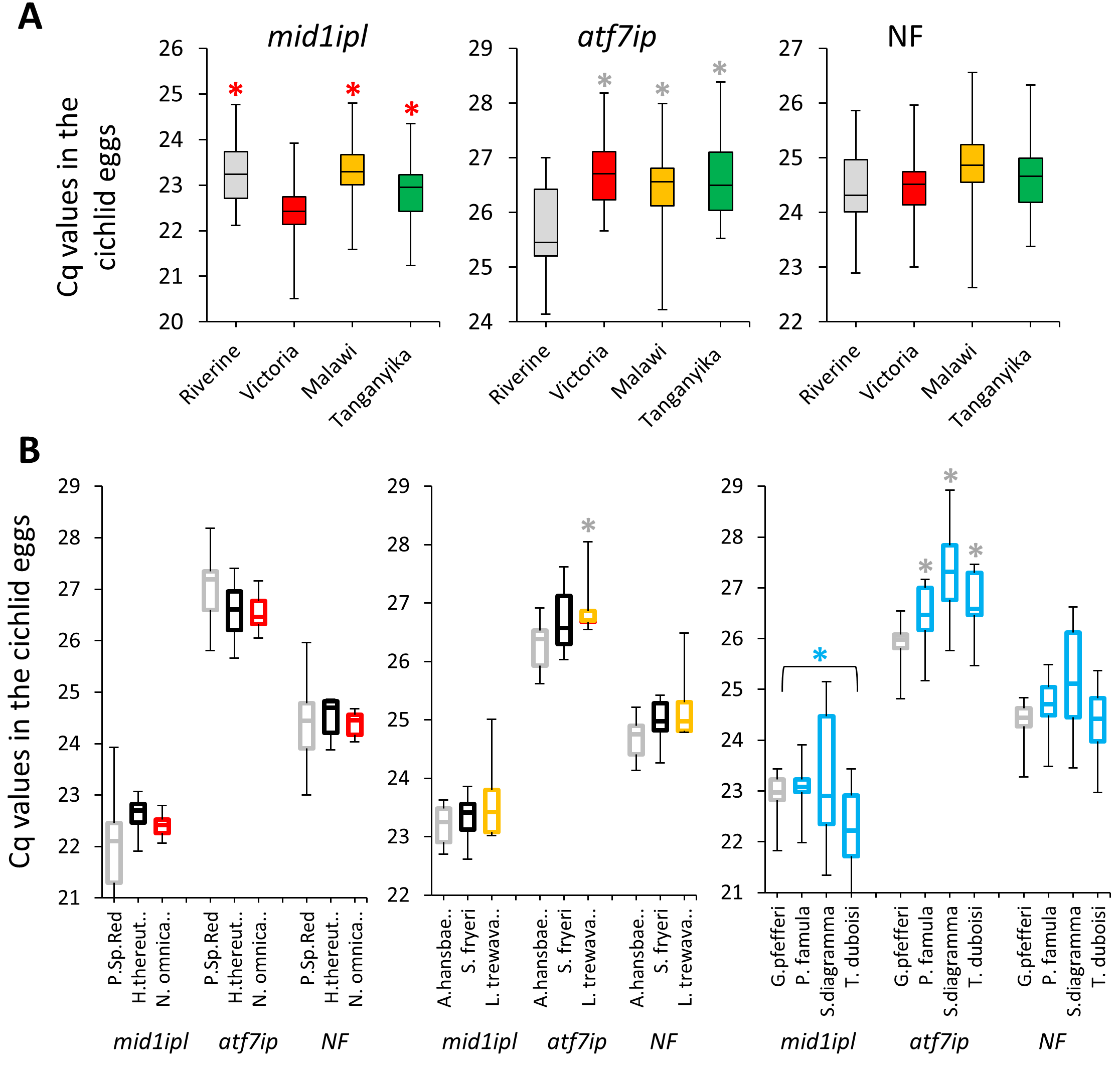

Supplement: Supplementary file 3 — Additional file 3: Figure 1. Differences of maternal mRNA abundance for mid1ip1 and atf7i, as well as geometric means their Cq Values (NF) in comparisons of the lakes (A) and the trophic niches (B). Asterisks above box plots indicate significantly elevated expression (P < 0.05) compared to the plots matching the colour of the asterisks. The middle line represents the median and boxes lower and upper limits indicate the 25/75 percentiles for each plot. [file 13227_2018_112_MOESM3_ESM.tif]
